# Development of a Cognitive Training Support Programme for prevention of dementia and cognitive decline in at-risk older adults

**DOI:** 10.3389/frdem.2024.1331741

**Published:** 2024-02-23

**Authors:** Celeste A. de Jager Loots, Geraint Price, Mariagnese Barbera, Anna Stigsdotter Neely, Hanna M. Gavelin, Jenni Lehtisalo, Tiia Ngandu, Alina Solomon, Francesca Mangialasche, Miia Kivipelto

**Affiliations:** ^1^Ageing Epidemiology Research Unit, School of Public Health, Imperial College London, London, United Kingdom; ^2^Department of Neurology, Institute of Clinical Medicine, University of Eastern Finland, Kuopio, Finland; ^3^Department of Social and Psychological Studies, Karlstad University, Karlstad, Sweden; ^4^Department of Health, Education and Technology, Luleå University of Technology, Luleå, Sweden; ^5^Department of Psychology, Umeå University, Umeå, Sweden; ^6^Population Health Unit, Finnish Institute for Health and Welfare, Helsinki, Finland; ^7^Division of Clinical Geriatrics, Center for Alzheimer Research, Department of Neurobiology, Care Sciences and Society, Karolinska Institutet, Stockholm, Sweden; ^8^FINGERS Brain Health Institute, Stockholm, Sweden; ^9^Theme Inflammation and Aging, Medical Unit Aging, Karolinska University Hospital, Stockholm, Sweden; ^10^Institute of Public Health and Clinical Nutrition, University of Eastern Finland, Kuopio, Finland

**Keywords:** Computerised Cognitive Training (CCT), lifestyle interventions, behaviour change, dementia risk factors, cognitive reserve, brain maintenance, ageing

## Abstract

**Background:**

Evidence for the beneficial effects of cognitive training on cognitive function and daily living activities is inconclusive. Variable study quality and design does not allow for robust comparisons/meta-analyses of different cognitive training programmes. Fairly low adherence to extended cognitive training interventions in clinical trials has been reported.

**Aims:**

The aim of further developing a Cognitive Training Support Programme (CTSP) is to supplement the Computerised Cognitive Training (CCT) intervention component of the multimodal Finnish Geriatric Intervention Study to Prevent Cognitive Impairment and Disability (FINGER), which is adapted to different cultural, regional and economic settings within the Word-Wide FINGERS (WW-FINGERS) Network. The main objectives are to improve adherence to cognitive training through a behaviour change framework and provide information about cognitive stimulation, social engagement and lifestyle risk factors for dementia.

**Methods:**

Six CTSP sessions were re-designed covering topics including (1) CCT instructions and tasks, (2) Cognitive domains: episodic memory, executive function and processing speed, (3) Successful ageing and compensatory strategies, (4) Cognitive stimulation and engagement, (5) Wellbeing factors affecting cognition (e.g., sleep and mood), (6) Sensory factors. Session content will be related to everyday life, with participant reflection and behaviour change techniques incorporated, e.g., strategies, goal-setting, active planning to enhance motivation, and adherence to the CCT and in relevant lifestyle changes.

**Conclusions:**

Through interactive presentations promoting brain health, the programme provides for personal reflection that may enhance capability, opportunity and motivation for behaviour change. This will support adherence to the CCT within multidomain intervention trials. Efficacy of the programme will be evaluated through participant feedback and adherence metrics.

## 1 Introduction

The Lancet Commission report on Dementia prevention, intervention and care, has estimated that 40% of dementia risk across the lifespan is due to modifiable factors, mainly based on data from studies in High Income Countries (HICs; Livingston et al., 2020). Overall dementia risk estimates are similar in Low and Middle-Income Countries (LMICs; Mukadam et al., 2019); however less childhood education, smoking, hypertension, obesity, and diabetes were more prevalent in LMICs than HICs. Risk factors related to mental engagement or cognitive stimulation include low levels of education and cognitive reserve, poor hearing, lack of social engagement and factors that affect social engagement such as depression. The report states that “although behaviour change is difficult, and some associations might not be purely causal, individuals have a huge potential to reduce their dementia risk.” The authors suggest a model where cognitive reserve can be increased or maintained by attending to hearing loss, attaining a high level of education, and maintaining a high level of social contact. Other risk factor modifications that, in this model, would feed into cognitive reserve include maintaining frequent exercise, avoiding excess alcohol consumption, and reducing the occurrence of depression. Those from more deprived environments or circumstances may benefit more from lifestyle interventions (Livingston et al., 2020).

Despite the recent promising results of three anti-amyloid monoclonal antibody trials (Cummings et al., 2023), disease-modifying pharmaceutical interventions are not yet widely available for prevention and, by targeting a single proteinopathy or biological mechanism alone, they may not be sufficient to prevent dementia (Ackley et al., 2021) nor to cure the underlying neurodegenerative diseases that cause it. Given the multifactorial aetiology of dementia, approaches targeting multiple risk factors at the same time may be needed for an optimal prevention effect (Kivipelto et al., 2018). Therefore, multimodal lifestyle interventions with behaviour change approaches have been tested in some large randomised clinical trials (RCTs) to help at-risk older adults in preventing dementia (Prevention of Dementia by Intensive Vascular care, PreDIVA (Moll van Charante et al., 2016); Multidomain Alzheimer Preventive Trial, MAPT (Andrieu et al., 2017), Maintain your Brain, MYB (Heffernan et al., 2019), Healthy Ageing Through Internet Counselling in the Elderly, HATICE (Richard et al., 2019). Among these relatively novel trials, the Finnish Geriatric Intervention study to Prevent Cognitive Impairment and Disability (FINGER) was the first RCT to show that a lifestyle intervention based on five components (diet, physical exercise, cognitive training, vascular and metabolic risk monitoring, and social engagement) could improve cognition and reduce the risk of cognitive impairment over 2 years (Ngandu et al., 2015). Interventions combining cognitive training with physical exercise (Shah et al., 2014; van het Reve, 2014), non-invasive brain stimulation (Krebs et al., 2023), and other interventions requiring behaviour change (Park et al., 2007) have also been tested showing improvement in cognitive and neural function.

The FINGER trial included series of cognitive training support sessions lead by psychologists to assist participants with navigating through the Computerised Cognitive Training (CCT) programme and understanding the tasks included. These group sessions provided information and discussions on age-related changes in cognition, memory strategies, and memory training in everyday activities, in order to increase participants' awareness and understanding on these topics (Kivipelto et al., 2013), as well as to enhance their engagement and adherence to the cognitive training intervention, done independently on a personal digital device.

Following the success of the FINGER trial, The World-Wide FINGERS (WW-FINGERS) network of trials investigating the effect of multimodal dementia prevention/risk reduction interventions was established (Kivipelto et al., 2020). The Networks' key aims are to: share knowledge and experiences; harmonise trials methodologies in different populations; combine data to generate robust evidence globally that will help advance the implementation of dementia prevention strategies. The Network (currently including 60+ countries) builds on the experiences and expands the work of the FINGER trial by tailoring and testing the FINGER model in different cultural, regional, and economic contexts. Like all other components of the FINGER intervention, a Cognitive Training Support Programme has served as a guide/template for the other studies in the Network and is now under further development to improve uptake, adherence, and effectiveness of the CCT programme.

### 1.1 Computerised Cognitive Training programmes

There has been a surge in development of commercial Computerised Cognitive Training (CCT) programmes to promote brain training for young and older people, and those with various neurological and mental health conditions. Cognitive training is defined as “specifically designed training programmes that provide guided practise on a standard set of cognitive tasks” (Kelly et al., 2014). They normally aim to improve neuroplasticity and cognitive function or skills in various cognitive domains including processing speed, working memory, executive function, visuospatial skills, verbal fluency, and episodic memory (Smith et al., 2009). Neuroplasticity is defined as the brain's ability to adapt to changes in the environment through modification, reorganisation, and creation of neural connexions (D'Antonio et al., 2019). Thus, intervention programmes attempt to generate neuroplasticity by exposing older adults to new learning environments. There are various theories on which cognitive training is based, including compensatory strategies involving transfer of activation from posterior brain regions to more frontal lobe regions (Crabtree, 2023). However, there is still little consensus on how to best develop CT programmes and on the evidence-base for its effectiveness, especially with regards to transfer of training to activities of daily living (Butler et al., 2018; Jaeggi et al., 2020).

### 1.2 Efficacy of Computerised Cognitive Training for cognitively healthy adults

The evidence of the beneficial effects of cognitive training on cognitive function and activities of daily living are inconclusive. CCT showed a small but statistically significant effect on global cognitive functioning for cognitively healthy older adults in meta-analyses of up to 90 trials compared with control performance (Lampit et al., 2014; Gavelin et al., 2020). However, a Cochrane review on the effectiveness of cognitive training for cognitively healthy adults in late life reported mostly low effect sizes of post-training cognitive performance in global cognition and domain-specific outcomes (Gates et al., 2019). They selected 8 RCTs (*n* = 1,138 participants) of 12–24 weeks duration and assessed those with active controls separately from those with passive controls. They concluded that study quality with low sample sizes, different outcome measures, length of training and other variables do not allow for robust comparisons of different cognitive training programmes or meta-analyses of combined data. The systematic review conducted in the context of the development of the first World Health Organisation (WHO) Guidelines for the Risk Reduction of Cognitive Decline and Dementia reported that the quality of the evidence supporting the efficacy of cognitive interventions in reducing the risk of cognitive decline was “very low to low” (Chowdhary et al., 2021).

An underlying aspect of current CCTs is that they are mostly conversions of neuropsychological or cognitive tests that are used in research studies and memory clinics to test cognitive function (Gross and Rebok, 2011). Mostly, training aimed at selected cognitive domains (e.g., mental processing speed) appears to transfer effects to post-training test performance in the same domain (processing speed) rather than more general cognition or daily function. The ecological validity and applicability to daily functional activity for those at risk of dementia or with a degree of cognitive impairment is therefore questionable. Some far transfer has been reported for aspects such as emotional state, diabetes management and depression, but there are few studies on far transfer of cognitive training for preventing cognitive decline in older adults. Tetlow and Edwards (2017) compared 14 CCTs used in RCTs, all adaptive and process-based programmes with either “no contact” (passive) or “active” (sham CCT or psychoeducation) control groups or both control groups in 18 studies. They found most evidence for effects on attention, processing speed and visuospatial memory (near transfer) while effects in all other cognitive domains including executive function and episodic memory were non-significant. They found some evidence of far transfer from meta-analysis of four studies in self-reported everyday function but not in performance-based everyday function or Instrumental Activities of Daily Living (IADL).

### 1.3 Adherence to cognitive training programmes

Fairly low adherence or compliance to extended cognitive training interventions in RCTs has been reported. For example, in the FINGER trial, it was reported that compliance was +/– 60%; however, this included participants who only completed the first session of cognitive training, while adherence was 47% when considering only individuals who attended over 50% of group sessions and completed over 50% of the computer training (Ngandu et al., 2015, 2022). The main driver of compliance was computer literacy (Turunen et al., 2019). As computer literacy has increased over the last decade, the expectation is that compliance might improve in further FINGER-like trials. The length, content variation and duration of cognitive training that is most beneficial to reducing cognitive decline or transferring to daily life has not yet been established. However, some research has shown that too long a duration of CCT has no more benefit than a shorter duration (Belleville et al., 2022).

Behaviour Change aspects of CCT programmes are a growing area of interest, and the factors most important for adherence and benefits still need further research.

### 1.4 Behaviour change theory

Various behaviour-change theories and frameworks have been described. The Capability, Opportunity, Motivation and Behaviour (COM-B) Behaviour Change Wheel (BCW) framework (Michie et al., 2011) was developed to improve on other frameworks that aim to influence public health. COM-B is a system involving three essential elements, Capability, Opportunity, and Motivation, which form the hub of a BCW, around which are nine intervention functions to change behaviour, either socially or individually. Capability includes the individual's psychological and physical capacity to engage in the activity concerned, with the necessary knowledge and skills. Motivation includes all the brain processes that energise and direct behaviour, including habitual processes, emotional responding, as well as analytical decision-making, not just goals and conscious decision-making. The BCW refers to both “reflective motivation” (based on positive or negative feelings relating to new information/knowledge and understanding) and “automatic motivation” (through associative learning via, e.g., imitation resulting in new habit formation). Opportunity can include physical or social changes to the environment that make the activity possible, or prompt it. Opportunity can influence motivation as can capability; enacting a behaviour can alter capability, motivation, and opportunity (Michie et al., 2011).

Researchers have also recommended use of structured frameworks in the development of CCT programmes to ensure that behaviour-change and efficacy of the programmes is optimised (Khaleghi et al., 2021; Peeters et al., 2023). Expectations and beliefs about the outcomes of CCT may affect training motivation and engagement (Tetlow and Edwards, 2017).

Peeters et al. (2023) reported on Behaviour Change Techniques (BCT) of CCT from 88 studies with cognitively healthy adults. There are 93 individual techniques in COM-B falling into 16 clusters or categories (Michie et al., 2015). Up to 34 BCTs were coded in the studies reviewed, with a median of 3. Commonly reported aspects included were items that were necessary for completion of the training programmes such as having instructions provided, adaptive or graded testing and feedback on outcomes of behaviour (amount of time in training or performance), however, these were not shown to significantly improve efficacy of the training on cognitive outcomes. The BCTs that had clinical relevance for efficacy included “monitoring of outcomes of behaviour without feedback by a person,” “feedback on outcomes of behaviour from a credible or known source”,” “information about social and environmental consequences.” “non-specific rewards” and “feedback on outcomes of behaviour” (delivered by a person) resulted in lower efficacy. BCTs that positively influenced adherence to CCT programmes included “self-monitoring of behaviour,” “monitoring of behaviour by others without feedback,” and “self-monitoring of outcomes of behaviour.” Studies using “graded tasks” had lower adherence. Self-reported barriers to adherence included excessive time commitment, health problems, lack of interest or motivation, holiday/travel, and lack of time (Peeters et al., 2023).

To bridge the gap in poor adherence and efficacy of CCT interventions in clinical trials, there is scope for add-on support encompassing behaviour change theory that provides information and education, compensatory strategies for successful ageing, and that enhances mental engagement and motivation. We have developed such a Cognitive Training Support Programme that includes aspects of the COM-B BCW described in this manuscript.

## 2 Aims

The aim of further developing the group sessions provided in the FINGER trial into a novel Cognitive Training Support Programme (CTSP) is to supplement and support the CCT used as a cognitive intervention component of the multimodal FINGER model, which is adapted to different cultural regional, and economic settings within the WW-FINGERS Network. We aim to describe the structure and content of the CTSP, its development and relation to COM-B. This CTSP is aimed at improving adherence to the cognitive training since the original FINGER trial (Turunen et al., 2019) showed relatively low adherence to CCT by older adults. The programme is intended to be delivered in small group settings. The group meeting format provides an opportunity for social interaction in addition to the skills-learning aspect of the programme. This approach will enhance engagement while providing the extra benefits of cognitive stimulation and psychological wellbeing (Dinius et al., 2023). It is also intended to enhance participants' awareness of real-world functional implications of cognitive decline and the effects of cognitive reserve, to optimise the possibility of impact on activities of daily living.

## 3 Methods

### 3.1 Study participants and recruitment

The original FINGER cognitive intervention was designed for older adults with no evidence of dementia or substantial cognitive impairment, but with some risk for dementia as assessed with the Cardiovascular Risk Factors, Ageing and Dementia (CAIDE) risk index (Kivipelto et al., 2006). Potential study participants should be screened for eligibility criteria including older age (~60 plus); performing slightly below the average or norm for age on cognitive testing; presence of modifiable risk factors for dementia, such as high Body Mass Index (BMI), high blood pressure, low levels of physical activity and with no conditions affecting effective engagement in the cognitive intervention. Study participants may be recruited from community-dwelling populations or registers of older people interested in taking part in healthy ageing and dementia prevention research. Based on the wider WW-FINGER framework and higher variability of settings, the intervention has been designed to be adjustable, e.g., to slightly different age groups, people with some level of cognitive impairment [e.g., Mild Cognitive Impairment (MCI)/prodromal Alzheimer's Disease] and adapted to different cultural, regional, and socio-economic contexts that reflect the diversity and variety of target populations within the global WW-FINGERS Network.

### 3.2 The Computerised Cognitive Training programme

The CCT used in the FINGER trial is intended for use in WW-FINGER dementia prevention multimodal intervention studies globally. This in-house CCT programme targets several critical age-sensitive cognitive functions focusing on processing speed, executive functions, working memory and episodic memory (Ronnlund et al., 2005; Salthouse, 2019) aiming to increase cognitive processing efficiency (Gavelin et al., 2015; Dinius et al., 2023). Training of a diverse set of cognitive processes that play key roles in cognition and brain functioning may have the potential to optimise generalisability of the intervention effect to non-trained tasks and everyday contexts (Schmiedek et al., 2010). The rationale for the design of this CCT programme is grounded on prior meta-analyses showing that multifactorial cognitive training programmes focusing on several age-sensitive cognitive functions may have benefits over single domain cognitive training programmes in terms of cognitive performance gains across domains (Lampit et al., 2014, 2019). The programme is based on cognitive tasks used in our prior research on healthy older and younger adults, persons with stress-related illnesses and Parkinson's Disease (Dahlin et al., 2008; Sandberg et al., 2014; Ngandu et al., 2015; Walton et al., 2017, 2020, 2021; Malmberg Gavelin et al., 2018), where results have shown improved performance in trained tasks as well as near transfer effects that are maintained over time. The programme includes six tasks, with two measuring episodic memory, using a verbal paired associates task and a location learning task, and one measuring working memory with a spatial-span task. Executive functions are addressed by two running span tasks that tap the updating function and one alternating run task addressing shifting. Processing speed training is incorporated into the shifting task. Moreover, the training tasks are adaptively adjusted to the performance level of each participant to tap into neuroplasticity by creating a mismatch between cognitive task demands and the cognitive resources (abilities) available to the person (Lovden et al., 2010).

### 3.3 Cognitive Training Support Programme

The support programme for group sessions for the CCT in WW-FINGER multimodal lifestyle intervention trials that our team, including neuropsychologists with RCT experience have developed includes a series of presentations that aim to keep participants engaged, motivated and compliant with the CCT schedule over a 2-year period. The sessions may be covered in the first few months of the intervention so that participants can become familiar with the CCT tasks and get assistance with any problems early in the trial to ensure confidence in continuing with the schedule. A motivational booster session may be included at the 12-month timepoint of the intervention to help maintain long term CCT adherence.

The cognitive stimulation support sessions that have been developed are described below.

#### 3.3.1 Group meetings

Study participants will be invited to a total of six group sessions led by trained psychologists. The first group session is focused on in-depth training for the CCT, so all participants will be enabled to engage independently in the CCT on a digital device. Subsequent sessions provide educational information on cognitive domain function, dementia risk factors, strategies for coping with age-related cognitive changes, importance of social engagement and topics for discussion.

Sessions 2–6 are structured as follows:

1. Brief cognitive training software refresh with question and answer (Q&A) time.
2. Main topic/theme of the meeting presented by a psychologist or trained team member.
3. Emerging tasks and topics for discussion.

### 3.4 Themes for six group sessions

#### 3.4.1 Cognitive training support session 1

This session will provide a brief introduction to the cognitive training programme and instructions for the use of the software. Detailed instructions for using a digital platform (tablet or computer) at home, logging into the training programme, reading and following the instructions and proceeding through the tasks will be given. Demonstrations of each of the training tasks will be given and the training plan outlined. Number of training sessions and time spent on each, the methods of automatically recording compliance by the programme, will be outlined. We will suggest that participants may want to keep their own training diary if it helps them to keep on track. It is important that study participants feel a sense of self-efficacy after this session and are enabled to train independently. In addition, once training has begun, participants will have the opportunity to ask questions at further group sessions on any problems they have experienced. A set of Frequently Asked Questions (FAQ) will be made available to cover common problems that may be encountered.

This first session will provide two aspects of the COM-B model, capability, and both physical and social opportunity. CCT performance is reported to be improved in a group setting (Lampit et al., 2014). Thus, the first session should enhance initial performance and motivation to proceed with training through the social support of the group and awareness that one is not alone in experiencing difficulties with technology or with the tasks. The group sessions should also provide a safe space for asking questions, and receiving help from a credible source, the trainer, face-to-face (Lampit et al., 2019). The CCT programme will also be put forward as a structured method to maintain cognitive stimulation or mental activity.

Providing increased understanding of what the CCT programme is aiming to achieve and how the tasks relate to everyday life will fit with COM-B theory in terms of increasing capability, through education, and in motivation, if participants understand that they have agency to maintain their neuroplasticity through new habit formation involved in the training.

#### 3.4.2 Cognitive training support session 2

The different types of cognitive domain functions and their meaning in the context of daily life.

In this session, the topic of cognitive domains such as episodic memory, working memory, attention and executive function will be explained in relation to the CCT tasks. Everyday examples of activities that use these cognitive domain functions will be given. For example, remembering a list of items needed at the grocery store or remembering an event that occurred the day before are examples of episodic memory. Participants may also relate to difficulties they have experienced in particular cognitive domains.

Transparency about what the CCT tasks involve may help alleviate frustration for participants who are struggling to achieve good results, as they may have a better understanding of why they find the tasks difficult. For example, it is well-known that mental and motor processing speed decline with age (Salthouse, 2019). If a task times out before one finishes and there is no explanation or feedback in the programme, participants may become frustrated at not being able to complete the task successfully. Thus, our in-house CCT does not time-out on tasks.

Topics of interest from the presentation about cognitive domain function may be discussed or about research behind the development of CCT programmes.

#### 3.4.3 Cognitive training support session 3

Cognition and ageing and compensatory mechanisms.

This session covers the topic of normal age-related changes in mental abilities. The concept of subjective memory complaints and how these are relevant to cognitive decline and dementia will be explained. The discussion will centre on the differences between changes commonly reported, including slowing down or losing attention as opposed to sudden changes (e.g., after an illness, stroke, or head injury), or consistent and progressive cognitive decline that is making life more difficult to manage independently. The aim will be to reduce anxiety about normal age-related changes and to clarify that dementia is not an inevitable consequence of ageing.

Elements of successful ageing and brain maintenance will be described through strategies such as the importance of selection (of what is important), optimisation (focusing on selected activities) and compensation (finding alternative ways of dealing with less important things or activities that have become difficult; Nyberg et al., 2012; Karlsen et al., 2022). Everyday examples may be related by the presenter and participants.

The concept of cognitive reserve and resilience to brain pathology will be introduced next, and that it may compensate and protect against losses that may occur through ageing and disease (Cabeza et al., 2018; Arenaza-Urquijo and Vemuri, 2020; Dinius et al., 2023). This level of reserve differs between people for example due to genetics, intelligence, education and lifetime experiences, thus degenerative brain changes will affect people differently in terms of the clinical symptoms they may present with such as cognitive decline (Stern et al., 2023).

The session will end with a time of reflection for participants to think of changes they have noticed in their thinking abilities with age (positive or negative), what changes they are intentionally able to compensate for and how, and what is important for them to focus on to keep feeling confident in their thinking and activities. This will involve some BCT in participants taking agency for what is important for them to focus on in terms of their cognitive abilities with some active planning and in increased understanding about the social and environmental consequences of normal ageing.

#### 3.4.4 Cognitive training support session 4

Cognitive stimulation, social engagement, related dementia risk factors and preventive activities.

This session is centred on the topic of cognitive stimulation to enhance neuroplasticity and engagement of all the senses. Cognitive stimulation refers to mentally engaging activities or exercises that challenge a person's ability to think. Cognitive stimulation aims to improve cognitive function through implicit learning tasks or activities to promote cognitive and social skills, while recognising the individual's own sense of self (Stine-Morrow et al., 2014; Stewart et al., 2017). Research on the impact of social engagement activities, networks, social support and relationships on cognitive function will be presented (Kelly et al., 2017). The concept of resilience will be introduced as a means to counteract effects of isolation and loneliness, and their impact on cognitive function (Tay and Lim, 2020).

A guided time of reflection will follow for participants to think of their personal situation and how this impacts their choices of stimulating activities. For example, those living alone may have different restrictions or availability in choices of activities to those living with a partner, spouse, or other family. The group will also be guided to think about what mental, social, and outdoor activities they enjoy doing and which activities they do alone or in company. There are various reasons to believe that brain training or other forms of cognitive stimulation will be enhanced if the activity is enjoyed rather than endured, due to the level of engagement experienced (Onafraychuk et al., 2021).

The session will end with time for the group to plan for incorporating new cognitively stimulating or challenging activities into their lives with some goal-setting. Lists of local organisations can be added to this session if participants want to take advantage of what is available for older people in their communities, for example, at museums, University of the Third Age (U3A) groups etc.

#### 3.4.5 Cognitive training support session 5

Wellbeing factors affecting brain function and cognition. Topics in this session include sleep, stress, anxiety and depression, food and mood. These factors can all affect cognitive function either short or long term and will be considered in terms of healthy ageing and prevention of dementia.

Sleep—the importance of sleep in amyloid clearance, long term memory storage and mood will be described followed by a definition of sleep quality (Kang et al., 2017; Casagrande et al., 2022). Alleviating anxiety about lack of sleep and advice on how to improve sleep quality will be discussed.

Stress – concepts of good and bad stress will be described in terms of the body's fight-flight mechanisms and hormones involved (McEwen, 2007; Dhabhar, 2014). What happens in the brain during short-term and chronic stress will be described in relation to memory.

Anxiety and Depression—these mood state concepts will be defined with some examples (Lindert et al., 2021). The benefits of some causes of anxiety as a protective instinct will be discussed. Some of the effects of anxiety on cognition will be described and disorders needing medical attention will be mentioned. As depression is often associated with stress and anxiety, these factors will be discussed together, with discussion of their causes and related symptoms.

Management of these three conditions in terms of BCT will be discussed with the group, giving time for participants to think of new patterns of behaviour that may be beneficial to themselves and in increasing their sense of self determination in enhancing their wellbeing.

Food and Mood—to discuss how certain foods and nutrients can affect mood. The benefits of a diet such as the Mediterranean Diet (Loughrey et al., 2017; Agarwal et al., 2023), shown in research studies to benefit cognition in older people, will be presented (Feart et al., 2010; Chou et al., 2019). The beneficial effects of certain nutrients on mood will be briefly described as will the effects of vitamin deficiencies.

Example of topics for discussion: Carb cravings, energy dips, comfort eating, caffeine stimulants- good or bad.

#### 3.4.6 Cognitive training support session 6

Sensory factors related to or affecting cognition including hearing, eye-sight, sense of smell, and dental health.

The importance of sensory input to understand our world and enhance mental activity and memory formation will be presented. Awareness about how intact senses can facilitate mental stimulation and independence in daily living will be discussed.

The group can contribute their own experiences related to sensory loss. Potential causes of sensory deprivation in eyesight, hearing, smell, taste and touch will be presented. Resulting effects of sensory loss such as social withdrawal and disengagement from activities such as reading, watching films, playing games, ability to drive can be discussed. Dangers of poor sense of smell and taste in terms of cooking, eating rotten food, detecting smoke or fire; poor sense of touch and peripheral neuropathy may be mentioned.

Research on sensory loss as signs of different types of dementia may be presented (Parkinson's and Lewy Body Disease and sense of smell; Dan et al., 2021).

Barriers to addressing sensory loss will be discussed including resistance to wearing hearing aids or glasses. Adaptations to sensory loss can be suggested including use of large print books, phone/TV screens, touch screen digital devices—phones, audio-loops, magnifying glasses and mirrors, and driving glasses. Examples such as having cataracts removed and the improvement noted can be shared by group members.

The positive use of pain to alert one to problems that need to be dealt with is a topic for discussion—to avoid bedsores, backpain, etc., one needs to respond to pain or discomfort signals by moving.

### 3.5 Inclusion of COM-B BCW aspects in the CSTP

To provide Capability we cover aspects of digital platform use associated with accessing the training, as well as examples of each of the CCT tasks with an explanation of the instructions to follow, the feedback to be received and ways to cope with any technical problems that arise. This will help participants to feel prepared for the training. Educational content in the sessions will cover the different cognitive domains being applied in each of the CCT tasks and related to everyday examples. Better understanding of brain function related to cognitive performance is intended to provide improved focus on the tasks. These two aspects will be covered in group sessions 1 and 2.

The Opportunity aspect of the COM-B model being implemented includes being in the active intervention group and having free access to the CCT for the duration of the trial, as well as support for those without home-based digital device availability. Participants will be encouraged to schedule regular weekly times for the training and to keep a training diary to track their progress as the self-monitoring BCT has previously been shown to increase adherence (Peeters et al., 2023). Adaptive testing to match participants' performance at each training session will provide the opportunity to be challenged at a level providing an optimal amount of challenge to enhance learning on the tasks. Although previous studies have not reported increased adherence to CCT with adaptive testing, this aspect needs further research.

Motivation is seen as a key component towards improved adherence to the CCT. Thus, a number of techniques will be implemented to enhance motivation, both intrinsic/automatic and extrinsic/reflective. These include Likert scales for self-monitoring of focus and motivation at the end of each training session; computer-based, graphically presented feedback on performance on each task at every session; a self-completed progress diary; face-to-face support with technical problems with the CCT during the group sessions and later via online support; knowledge that compliance will be automatically tracked at every training session completed. The educational components of the CSTP may also increase motivation in terms of participant's beliefs about the consequences of adhering to the CCT, the relevance of the training to cognitive functions affecting everyday life and in increasing agency for healthier ageing. The knowledge that study investigators will be tracking adherence via recorded logons and completion of CCT tasks may also be a motivator for those who respond to external feedback (Peeters et al., 2023).

## 4 Results and feedback

The Cognitive Training Support Programme will be completed with a feedback questionnaire for reflection of responses to the programme. Participant evaluation of items including enjoyment, benefits, motivation, relevance, ease of comprehension, engagement, and other aspects of the programme will be sought for a qualitative analysis of its effectiveness. Feedback on the CCT in terms of ease of navigation and technical issues, engagement and adherence will also be surveyed.

A cognitive and social activity questionnaire, as used in the FINGER trial, will collect average frequency of reading, crosswords, writing, games, listening or playing music, communal activities or participation in societies, studying, handicrafts, gardening, baby-sitting, and voluntary work on a 7-point Likert scale with alternatives from daily to never. The results will be translated into weekly activities and summarised. In addition, computer use will be asked separately (yes/no), and computer usage provided as one additional activity (corresponding to once per week).

Evaluating the effectiveness of the CTSP can be correlated with measures of adherence to the CCT. The Likert scales added to the CCT on motivation and attention will also be informative and will be important in understanding fluctuations in performance over time. In addition, when used in a study with cognitive outcomes, change in cognitive performance from the beginning to end of the programme would be the best quantitative measure of efficacy of the intervention. A pilot trial of the Cognitive Intervention (CCT plus Support programme) is desirable before adding it to a multimodal intervention trial.

## 5 Discussion

Cognitive reserve, or resilience against cognitive decline may involve increased connectivity in frontal and temporal brain regions as well as good blood flow to the brain via metabolic mechanisms. Thus, building cognitive reserve through new neural network formation and supporting brain maintenance are crucial components of non-pharmacological approaches to promoting cognitive and psychological function in older age and in early dementia (Gajewski and Falkenstein, 2016; Allen et al., 2020; Dinius et al., 2023). Age-related cognitive decline can be reduced through interventions including cognitive stimulation, social engagement and mental wellbeing (Wilson et al., 2012). The engagement model strives to embed the participant in complex environments that are socially and intellectually enriching, as well as challenging, as they require a diverse range of abilities rather than mastery of one skill (Stine-Morrow et al., 2014). Cognitive stimulation is a broader concept than cognitive training but can also encompass structured cognitive training. While Cognitive Stimulation Therapy (CST) programmes have been developed for people living with dementia and are recommended as a non-pharmacological intervention (Gonzalez-Moreno et al., 2022a,b), CST for cognitively healthy or mild cognitive impairment is not readily available.

However, as the evidence for efficacy and adherence to cognitive training, especially for long term clinical trials, is variable, further research is needed, both in CCT programmes and in the BCT incorporated in them. Many CCT programmes are merely adaptations of cognitive tests in different domains, thus the ecological validity for transfer to daily life is limited. There have been novel proposals to develop more 3-dimensional CCTs, serious video games and immersive sensory stimulation modules to increase participant engagement and for better transfer of training effects to long term gains in functional activity (Mishra et al., 2016; Sokolov et al., 2020). However, a video-gaming programme trialled for effects on cognitive performance found that older adults do not enjoy the same sorts of video games as younger people, such as Super Mario and action games with shooting of targets. Participants reported that the games were more boring than reading and other daily hobbies that were cognitively stimulating. Thus, motivation was not well-achieved. However, there are other studies with video-gaming showing some evidence of positive effects on cognitive function (Anguera et al., 2021; Gavelin et al., 2021). Sikkes et al. (2021) concluded that what is required is greater understanding of how to develop more personalised CCT treatments that can be better integrated with everyday life and meaningful everyday activities, so that ongoing engagement is more likely and that transfer of gains from performance on standard tests of cognition to relevant functional domains is enabled.

Our novel approach to overcome the barriers to engagement and adherence to CCT was to further develop the group sessions in FINGER into a Cognitive Training Support Programme (CTSP) to be led by trained psychologists, as described. Studies have shown that supplementing CCT with self-management programmes may produce greater benefits in cognition relative to cognitive training alone (Hertzog et al., 2020). The CTSP will provide participating older adults with information about cognitive function and how it relates to lifestyle choices, abilities, social setting, mental health and sensory capabilities. Thus, improved awareness of the benefits of cognitive stimulation and the ability to address modifiable lifestyle risk factors for dementia such as poor sleep, hearing, etc., aims to enhance the efficacy of both the CCT and the CTSP.

Through interactive presentations and discussion of various strategies for successful ageing and to prevent cognitive decline to dementia, the programme provides time for personal reflection, goal setting, and active planning that may enhance behaviour change and influence participant motivation, engagement and expectations of adhering to the in-house CCT intervention described. The COM-B behaviour-change aspects incorporated both in the CCT and the CSTP are based on results from previous analyses of aspects that show some efficacy in improving engagement and adherence to cognitive training interventions (Peeters et al., 2023). The development of this Cognitive Training Support Programme is for delivery in-person in group settings- another BCT that has shown positive effects in CCT efficacy (Lampit et al., 2014). However, it is adaptable for online use for small groups where interaction is possible. We have produced it in a series of slide presentations, but these may be converted to recorded video presentations. Discussion and reflection time has been built into the time-frame of the sessions. The design of the programme aims to be flexible enough for cross-cultural adaptation for global use either with CCT or “pen and paper” cognitive training. Some of the BCT and concepts can still be used in conjunction with Non-Computerised Cognitive Training. However, digital transition is happening rapidly in several LMIC, with the advantage of increased scalability and reduced costs for studies. Further modifications may be made to the CTSP to introduce more interactive content with co-design through participant engagement initiatives.

## 6 Conclusions

It was deemed appropriate to supplement the CCT used in FINGER with a newly expanded CTSP for group sessions due to reports of variable and low efficacy on cognitive performance and adherence to cognitive training programmes. In addition, BCTs incorporated into CCTs have shown some clinical relevance, but more evidence is needed for their efficacy on cognition. Thus, a support programme promoting brain health through cognitive stimulation and social engagement, as well as providing information about preventing dementia risk related to modifiable lifestyle factors is hoped to prove effective in enhancing both adherence and efficacy of CCTs. The programme provides for personal reflection that may enhance capability, opportunity, and motivation for behaviour change. The incorporation of multiple BCTs into the CTSP will allow for further investigation of their usefulness in this context.

## Data availability statement

The original contributions presented in the study are included in the article/supplementary material, further inquiries can be directed to the corresponding author.

## Author contributions

CL: Conceptualisation, Investigation, Methodology, Writing – original draft, Writing – review & editing. GP: Conceptualisation, Methodology, Writing – review & editing. MB: Methodology, Project administration, Writing – original draft, Writing – review & editing. AN: Investigation, Methodology, Writing – original draft, Writing – review & editing. HG: Methodology, Writing – review & editing. JL: Methodology, Writing – review & editing. TN: Methodology, Writing – review & editing, Funding acquisition. AS: Funding acquisition, Writing – review & editing. FM: Funding acquisition, Investigation, Writing – review & editing. MK: Funding acquisition, Writing – review & editing.

## References

[B1] Ackley S. F. Zimmerman S. C. Brenowitz W. D. Tchetgen Tchetgen E. J. Gold A. L. Manly J. J. . (2021). Effect of reductions in amyloid levels on cognitive change in randomized trials: instrumental variable meta-analysis. Br. Med. J. 372:n156. 10.1136/bmj.n156 33632704 PMC7905687

[B2] Agarwal P. Leurgans S. E. Agrawal S. Aggarwal N. T. Cherian L. J. James B. D. . (2023). Association of mediterranean-DASH intervention for neurodegenerative delay and mediterranean diets with Alzheimer disease pathology. Neurology 100, e2259–e68. 10.1212/WNL.0000000000207176 36889921 PMC10259273

[B3] Allen S. F. Gilbody S. Atkin K. van der Feltz-Cornelis C. (2020). The associations between loneliness, social exclusion and pain in the general population: a *N* = 502,528 cross-sectional UK Biobank study. J. Psychiatr. Res. 130, 68–74. 10.1016/j.jpsychires.2020.06.028 32791383

[B4] Andrieu S. Guyonnet S. Coley N. Cantet C. Bonnefoy M. Bordes S. . (2017). Effect of long-term omega 3 polyunsaturated fatty acid supplementation with or without multidomain intervention on cognitive function in elderly adults with memory complaints (MAPT): a randomised, placebo-controlled trial. Lancet Neurol. 16, 377–389. 10.1016/S1474-4422(17)30040-6 28359749

[B5] Anguera J. A. Schachtner J. N. Simon A. J. Volponi J. Javed S. Gallen C. L. . (2021). Long-term maintenance of multitasking abilities following video game training in older adults. Neurobiol. Aging. 103, 22–30. 10.1016/j.neurobiolaging.2021.02.023 33789209

[B6] Arenaza-Urquijo E. M. Vemuri P. (2020). Improving the resistance and resilience framework for aging and dementia studies. Alzheimer's Res. Ther. 12:41. 10.1186/s13195-020-00609-2 32290864 PMC7158381

[B7] Belleville S. Cloutier S. Mellah S. Willis S. Vellas B. Andrieu S. . (2022). Is more always better? Dose effect in a multidomain intervention in older adults at risk of dementia. Alzheimer's Dement. 18, 2140–2150. 10.1002/alz.12544 35049127 PMC9786573

[B8] Butler M. McCreedy E. Nelson V. A. Desai P. Ratner E. Fink H. A. . (2018). Does cognitive training prevent cognitive decline? a systematic review. Ann. Intern. Med. 168, 63–68. 10.7326/M17-1531 29255842

[B9] Cabeza R. Albert M. Belleville S. Craik F. I. M. Duarte A. Grady C. L. . (2018). Maintenance, reserve and compensation: the cognitive neuroscience of healthy ageing. Nat. Rev. Neurosci. 19, 701–710. 10.1038/s41583-018-0068-2 30305711 PMC6472256

[B10] Casagrande M. Forte G. Favieri F. Corbo I. (2022). Sleep quality and aging: a systematic review on healthy older people, mild cognitive impairment and Alzheimer's disease. Int. J. Environ. Res. Publ. Health 19:148457. 10.3390/ijerph19148457 35886309 PMC9325170

[B11] Chou Y. C. Lee M. S. Chiou J. M. Chen T. F. Chen Y. C. Chen J. H. . (2019). Association of diet quality and vegetable variety with the risk of cognitive decline in Chinese older adults. Nutrients 11:71666. 10.3390/nu11071666 31330854 PMC6682985

[B12] Chowdhary N. Barbui C. Anstey K. J. Kivipelto M. Barbera M. Peters R. . (2021). Reducing the risk of cognitive decline and dementia: WHO recommendations. Front. Neurol. 12:765584. 10.3389/fneur.2021.765584 35082745 PMC8784726

[B13] Crabtree A. (2023). “Is there anything I can do to train my memory?” The rationale and evidence behind cognitive training as an intervention to promote healthy ageing. FPOP Bullet. 1, 68–73. 10.53841/bpsfpop.2023.1.163.68

[B14] Cummings J. Zhou Y. Lee G. Zhong K. Fonseca J. Cheng F. . (2023). Alzheimer's disease drug development pipeline: 2023. Alzheimer's Dement. 9:e12385. 10.1002/trc2.12385 PMC10210334 37251912

[B15] Dahlin E. Nyberg L. Backman L. Neely A. S. (2008). Plasticity of executive functioning in young and older adults: immediate training gains, transfer, and long-term maintenance. Psychol. Aging 23, 720–730. 10.1037/a0014296 19140643

[B16] Dan X. Wechter N. Gray S. Mohanty J. G. Croteau D. L. Bohr V. A. . (2021). Olfactory dysfunction in aging and neurodegenerative diseases. Ageing Res. Rev. 70:101416. 10.1016/j.arr.2021.101416 34325072 PMC8373788

[B17] D'Antonio J. Simon-Pearson L. Goldberg T. Sneed J. R. Rushia S. Kerner N. . (2019). Cognitive training and neuroplasticity in mild cognitive impairment (COG-IT): protocol for a two-site, blinded, randomised, controlled treatment trial. Br. Med. J. Open 9:e028536. 10.1136/bmjopen-2018-028536 31471436 PMC6720324

[B18] Dhabhar F. S. (2014). Effects of stress on immune function: the good, the bad, and the beautiful. Immunol. Res. 58, 193–210. 10.1007/s12026-014-8517-0 24798553

[B19] Dinius C. J. Pocknell C. E. Caffrey M. P. Roche R. A. P. (2023). Cognitive interventions for memory and psychological well-being in aging and dementias. Front. Psychol. 14:1070012. 10.3389/fpsyg.2023.1070012 36818134 PMC9932670

[B20] Feart C. Samieri C. Barberger-Gateau P. (2010). Mediterranean diet and cognitive function in older adults. Curr. Opin. Clin. Nutr. Metab. Care. 13, 14–18. 10.1097/MCO.0b013e3283331fe4 19834324 PMC2997798

[B21] Gajewski P. D. Falkenstein M. (2016). Physical activity and neurocognitive functioning in aging - a condensed updated review. Eur. Rev. Aging Phys. Act. 13:1. 10.1186/s11556-016-0161-3 26865880 PMC4748322

[B22] Gates N. J. Rutjes A. W. Di Nisio M. Karim S. Chong L. Y. March E. . (2019). Computerised cognitive training for maintaining cognitive function in cognitively healthy people in late life. Cochr. Datab. Syst. Rev. 3:CD012277. 10.1002/14651858.CD012277.pub2 30864187 PMC6414816

[B23] Gavelin H. M. Boraxbekk C.-. J Stenlund T. Järvholm L. S. Neely A. S. (2015). Effects of a process-based cognitive training intervention for patients with stress-related exhaustion. Stress 18, 578–588. 10.3109/10253890.2015.1064892 26305186

[B24] Gavelin H. M. Dong C. Minkov R. Bahar-Fuchs A. Ellis K. A. Lautenschlager N. T. . (2021). Combined physical and cognitive training for older adults with and without cognitive impairment: a systematic review and network meta-analysis of randomized controlled trials. Ageing Res. Rev. 66:101232. 10.1016/j.arr.2020.101232 33249177

[B25] Gavelin H. M. Lampit A. Hallock H. Sabates J. Bahar-Fuchs A. (2020). Cognition-oriented treatments for older adults: a systematic overview of systematic reviews. Neuropsychol. Rev. 30, 167–193. 10.1007/s11065-020-09434-8 32266520 PMC7305099

[B26] Gonzalez-Moreno J. Satorres E. Soria-Urios G. Melendez J. C. (2022a). Cognitive stimulation program presented through new technologies in a group of people with moderate cognitive impairment. J. Alzheimer's Dis. 88, 513–519. 10.3233/JAD-220245 35662124

[B27] Gonzalez-Moreno J. Satorres E. Soria-Urios G. Melendez J. C. (2022b). Cognitive stimulation in moderate Alzheimer's disease. J. Appl. Gerontol. 41, 1934–1941. 10.1177/07334648221089283 35621327

[B28] Gross A. L. Rebok G. W. (2011). Memory training and strategy use in older adults: results from the ACTIVE study. Psychol. Aging 26, 503–517. 10.1037/a0022687 21443356 PMC3573329

[B29] Heffernan M. Andrews G. Fiatarone Singh M. A. Valenzuela M. Anstey K. J. Maeder A. J. . (2019). Maintain your brain: protocol of a 3-year randomized controlled trial of a personalized multi-modal digital health intervention to prevent cognitive decline among community dwelling 55 to 77 year olds. J. Alzheimer's Dis. 70, S221–S37. 10.3233/JAD-180572 30475762 PMC6700632

[B30] Hertzog C. Pearman A. Lustig E. Hughes M. (2020). Fostering self-management of everyday memory in older adults: a new intervention approach. Front. Psychol. 11:560056. 10.3389/fpsyg.2020.560056 33488441 PMC7817715

[B31] Jaeggi S. M. Buschkuehl M. Parlett-Pelleriti C. M. Moon S. M. Evans M. Kritzmacher A. . (2020). Investigating the effects of spacing on working memory training outcome: a randomized, controlled, multisite trial in older adults. J. Gerontol. B Psychol. Sci. Soc. Sci. 75, 1181–1192. 10.1093/geronb/gbz090 31353413 PMC7265810

[B32] Kang D. W. Lee C. U. Lim H. K. (2017). Role of sleep disturbance in the trajectory of Alzheimer's disease. Clin. Psychopharmacol. Neurosci. 15, 89–99. 10.9758/cpn.2017.15.2.89 28449556 PMC5426492

[B33] Karlsen I. L. Borg V. Meng A. (2022). Exploring the use of selection, optimization, and compensation strategies beyond the individual level in a workplace context—a qualitative case study. Front. Psychol. 13:832241. 10.3389/fpsyg.2022.832241 35222210 PMC8866242

[B34] Kelly M. E. Duff H. Kelly S. McHugh Power J. E. Brennan S. Lawlor B. A. . (2017). The impact of social activities, social networks, social support and social relationships on the cognitive functioning of healthy older adults: a systematic review. Syst. Rev. 6:259. 10.1186/s13643-017-0632-2 29258596 PMC5735742

[B35] Kelly M. E. Loughrey D. Lawlor B. A. Robertson I. H. Walsh C. Brennan S. . (2014). The impact of cognitive training and mental stimulation on cognitive and everyday functioning of healthy older adults: a systematic review and meta-analysis. Ageing Res. Rev. 15, 28–43. 10.1016/j.arr.2014.02.004 24607830

[B36] Khaleghi A. Aghaei Z. Mahdavi M. A. A. (2021). Gamification framework for cognitive assessment and cognitive training: qualitative study. JMIR Serious Games 9:e21900. 10.2196/21900 33819164 PMC8170558

[B37] Kivipelto M. Mangialasche F. Ngandu T. (2018). Lifestyle interventions to prevent cognitive impairment, dementia and Alzheimer disease. Nat. Rev. Neurol. 14, 653–666. 10.1038/s41582-018-0070-3 30291317

[B38] Kivipelto M. Mangialasche F. Snyder H. M. Allegri R. Andrieu S. Arai H. . (2020). World-Wide FINGERS Network: a global approach to risk reduction and prevention of dementia. Alzheimer's Dement. 16, 1078–1094. 10.1002/alz.12123 32627328 PMC9527644

[B39] Kivipelto M. Ngandu T. Laatikainen T. Winblad B. Soininen H. Tuomilehto J. . (2006). Risk score for the prediction of dementia risk in 20 years among middle aged people: a longitudinal, population-based study. Lancet Neurol. 5, 735–741. 10.1016/S1474-4422(06)70537-3 16914401

[B40] Kivipelto M. S. A. Ahtiluoto S. Ngandu T. Lehtisalo J. Antikainen R. Bäckman L. . (2013). The Finnish geriatric intervention study to prevent cognitive impairment and disability (FINGER): study design and progress. Alzheimer's Dement. 9, 657–665. 10.1016/j.jalz.2012.09.012 23332672

[B41] Krebs C. Peter J. Brill E. Kloppel S. Brem A. K. (2023). The moderating effects of sex, age, and education on the outcome of combined cognitive training and transcranial electrical stimulation in older adults. Front. Psychol. 14:1243099. 10.3389/fpsyg.2023.1243099 37809311 PMC10556861

[B42] Lampit A. Hallock H. Valenzuela M. (2014). Computerized cognitive training in cognitively healthy older adults: a systematic review and meta-analysis of effect modifiers. PLoS Med. 11:e1001756. 10.1371/journal.pmed.1001756 25405755 PMC4236015

[B43] Lampit A. Heine J. Finke C. Barnett M. H. Valenzuela M. Wolf A. . (2019). Computerized cognitive training in multiple sclerosis: a systematic review and meta-analysis. Neurorehabil. Neural Repair 33, 695–706. 10.1177/1545968319860490 31328637

[B44] Lindert J. Paul K. C. Lachman M. E. Ritz B. Seeman T. E. (2021). Depression-, anxiety-, and anger and cognitive functions: findings from a longitudinal prospective study. Front. Psychiatry 12:665742. 10.3389/fpsyt.2021.665742 34421666 PMC8377351

[B45] Livingston G. Huntley J. Sommerlad A. Ames D. Ballard C. Banerjee S. . (2020). Dementia prevention, intervention, and care: 2020 report of the Lancet Commission. Lancet 396, 413–446. 10.1016/S0140-6736(20)30367-6 32738937 PMC7392084

[B46] Loughrey D. G. Lavecchia S. Brennan S. Lawlor B. A. Kelly M. E. (2017). The impact of the mediterranean diet on the cognitive functioning of healthy older adults: a systematic review and meta-analysis. Adv. Nutr. 8, 571–586. 10.3945/an.117.015495 28710144 PMC5502874

[B47] Lovden M. Backman L. Lindenberger U. Schaefer S. Schmiedek F. (2010). A theoretical framework for the study of adult cognitive plasticity. Psychol. Bull. 136, 659–676. 10.1037/a0020080 20565172

[B48] Malmberg Gavelin H. Eskilsson T. Boraxbekk C. J. Josefsson M. Stigsdotter Neely A. Slunga Jarvholm L. . (2018). Rehabilitation for improved cognition in patients with stress-related exhaustion disorder: RECO—a randomized clinical trial. Stress 21, 279–291. 10.1080/10253890.2018.1461833 29693483

[B49] McEwen B. S. (2007). Physiology and neurobiology of stress and adaptation: central role of the brain. Physiol. Rev. 87, 873–904. 10.1152/physrev.00041.2006 17615391

[B50] Michie S. van Stralen M. M. West R. (2011). The behaviour change wheel: a new method for characterising and designing behaviour change interventions. Implement. Sci. 6:42. 10.1186/1748-5908-6-42 21513547 PMC3096582

[B51] Michie S. Wood C. E. Johnston M. Abraham C. Francis J. J. Hardeman W. . (2015). Behaviour change techniques: the development and evaluation of a taxonomic method for reporting and describing behaviour change interventions (a suite of five studies involving consensus methods, randomised controlled trials and analysis of qualitative data). Health Technol. Assess. 19, 1–188. 10.3310/hta19990 PMC4781650 26616119

[B52] Mishra J. Anguera J. A. Gazzaley A. (2016). Video games for neuro-cognitive optimization. Neuron 90, 214–218. 10.1016/j.neuron.2016.04.010 27100194

[B53] Moll van Charante E. P. Richard E. Eurelings L. S. van Dalen J. W. Ligthart S. A. van Bussel E. F. . (2016). Effectiveness of a 6-year multidomain vascular care intervention to prevent dementia (preDIVA): a cluster-randomised controlled trial. Lancet 388, 797–805. 10.1016/S0140-6736(16)30950-3 27474376

[B54] Mukadam N. Sommerlad A. Huntley J. Livingston G. (2019). Population attributable fractions for risk factors for dementia in low-income and middle-income countries: an analysis using cross-sectional survey data. Lancet Glob. Health 7, e596–e603. 10.1016/S2214-109X(19)30074-9 31000129 PMC7617123

[B55] Ngandu T. Lehtisalo J. Korkki S. Solomon A. Coley N. Antikainen R. . (2022). The effect of adherence on cognition in a multidomain lifestyle intervention (FINGER). Alzheimer's Dement. 18, 1325–1334. 10.1002/alz.12492 34668644

[B56] Ngandu T. Lehtisalo J. Solomon A. Levalahti E. Ahtiluoto S. Antikainen R. . (2015). A 2 year multidomain intervention of diet, exercise, cognitive training, and vascular risk monitoring versus control to prevent cognitive decline in at-risk elderly people (FINGER): a randomised controlled trial. Lancet 385, 2255–2263. 10.1016/S0140-6736(15)60461-5 25771249

[B57] Nyberg L. Lovden M. Riklund K. Lindenberger U. Backman L. (2012). Memory aging and brain maintenance. Trends Cogn. Sci. 16, 292–305. 10.1016/j.tics.2012.04.005 22542563

[B58] Onafraychuk D. Sanders E. C. Harrell E. R. Boot W. R. (2021). Exploring individuals' willingness to engage in interventions to improve cognitive health and prolong late-life independence: an extension of Harrell, Kmetz, and Boot (2019). J. Cogn. Enhanc. 5, 259–265. 10.1007/s41465-020-00197-x 34485809 PMC8415010

[B59] Park D. C. Gutchess A. H. Meade M. L. Stine-Morrow E. A. (2007). Improving cognitive function in older adults: nontraditional approaches. J. Gerontol. B Psychol. Sci. Soc. Sci. 62, 45–52. 10.1093/geronb/62.special_issue_1.45 17565164

[B60] Peeters G. Black I. L. Gomersall S. R. Fritschi J. Sweeney A. Guedes A. . (2023). Behaviour change techniques in computerized cognitive training for cognitively healthy older adults: a systematic review. Neuropsychol. Rev. 33, 238–254. 10.1007/s11065-022-09537-4 35157209 PMC9998598

[B61] Richard E. Moll van Charante E. P. Hoevenaar-Blom M. P. Coley N. Barbera M. van der Groep A. . (2019). Healthy ageing through internet counselling in the elderly (HATICE): a multinational, randomised controlled trial. Lancet Digit. Health 1, e424–e34. 10.1016/S2589-7500(19)30153-0 33323224

[B62] Ronnlund M. Nyberg L. Backman L. Nilsson L. G. (2005). Stability, growth, and decline in adult life span development of declarative memory: cross-sectional and longitudinal data from a population-based study. Psychol. Aging 20, 3–18. 10.1037/0882-7974.20.1.3 15769210

[B63] Salthouse T. A. (2019). Trajectories of normal cognitive aging. Psychol. Aging 34, 17–24. 10.1037/pag0000288 30211596 PMC6367038

[B64] Sandberg P. Ronnlund M. Nyberg L. Stigsdotter Neely A. (2014). Executive process training in young and old adults. Neuropsychol. Dev. Cogn. B Aging Neuropsychol. Cogn. 21, 577–605. 10.1080/13825585.2013.839777 24148093

[B65] Schmiedek F. Lovden M. Lindenberger U. (2010). Hundred days of cognitive training enhance broad cognitive abilities in adulthood: findings from the COGITO study. Front. Aging Neurosci. 2:27. 10.3389/fnagi.2010.00027 20725526 PMC2914582

[B66] Shah T. Verdile G. Sohrabi H. Campbell A. Putland E. Cheetham C. . (2014). A combination of physical activity and computerized brain training improves verbal memory and increases cerebral glucose metabolism in the elderly. Transl. Psychiatry 4:e487. 10.1038/tp.2014.122 25463973 PMC4270308

[B67] Sikkes S. A. M. Tang Y. Jutten R. J. Wesselman L. M. P. Turkstra L. S. Brodaty H. . (2021). Toward a theory-based specification of non-pharmacological treatments in aging and dementia: focused reviews and methodological recommendations. Alzheimer's Dement. 17, 255–270. 10.1002/alz.12188 33215876 PMC7970750

[B68] Smith G. E. Housen P. Yaffe K. Ruff R. Kennison R. F. Mahncke H. W. . (2009). A cognitive training program based on principles of brain plasticity: results from the Improvement in Memory with Plasticity-based Adaptive Cognitive Training (IMPACT) study. J. Am. Geriatr. Soc. 57, 594–603. 10.1111/j.1532-5415.2008.02167.x 19220558 PMC4169294

[B69] Sokolov A. A. Collignon A. Bieler-Aeschlimann M. (2020). Serious video games and virtual reality for prevention and neurorehabilitation of cognitive decline because of aging and neurodegeneration. Curr. Opin. Neurol. 33, 239–248. 10.1097/WCO.0000000000000791 32073439

[B70] Stern Y. Albert M. Barnes C. A. Cabeza R. Pascual-Leone A. Rapp P. R. . (2023). A framework for concepts of reserve and resilience in aging. Neurobiol. Aging 124, 100–103. 10.1016/j.neurobiolaging.2022.10.015 36653245 PMC10424718

[B71] Stewart D. B. Berg-Weger M. Tebb S. Sakamoto M. Roselle K. Downing L. . (2017). Making a difference: a study of cognitive stimulation therapy for persons with dementia. J. Gerontol. Soc. Work 60, 300–312. 10.1080/01634372.2017.1318196 28409672

[B72] Stine-Morrow E. A. L. Payne B. R. Roberts B. W. Kramer A. F. Morrow D. G. Payne L. . (2014). Training versus engagement as paths to cognitive enrichment with aging. Psychol. Aging 29, 891–906. 10.1037/a0038244 25402337 PMC4361254

[B73] Tay P. K. C. Lim K. K. (2020). Psychological resilience as an emergent characteristic for well-being: a pragmatic view. Gerontology 66, 476–483. 10.1159/000509210 32784301

[B74] Tetlow A. M. Edwards J. D. (2017). Systematic literature review and meta-analysis of commercially available computerized cognitive training among older adults. J. Cogn. Enhanc. 1, 559–575. 10.1007/s41465-017-0051-2

[B75] Turunen M. Hokkanen L. Backman L. Stigsdotter-Neely A. Hanninen T. Paajanen T. . (2019). Computer-based cognitive training for older adults: determinants of adherence. PLoS ONE 14:e0219541. 10.1371/journal.pone.0219541 31291337 PMC6620011

[B76] van het Reve E. B. E. (2014). Strength-balance supplemented with computerized cognitive training to improve dual task gait and divided attention in older adults: a multicenter randomized-controlled trial. BMC Geriatr. 14:134. 10.1186/1471-2318-14-134 25511081 PMC4293005

[B77] Walton C. C. Naismith S. L. Lampit A. Mowszowski L. Lewis S. J. (2017). Cognitive training in Parkinson's disease. Neurorehabil. Neural Repair 31, 207–216. 10.1177/1545968316680489 27899737

[B78] Walton L. Domellof M. E. Astrom A. N. Elowson A. Neely A. S. (2021). Digital dance for people with Parkinson's disease during the COVID-19 pandemic: a feasibility study. Front. Neurol. 12:743432. 10.3389/fneur.2021.743432 35185746 PMC8850348

[B79] Walton L. Domellof M. E. Boraxbekk C. J. Domellof E. Ronnqvist L. Backstrom D. . (2020). The effects of working memory updating training in Parkinson's disease: a feasibility and single-subject study on cognition, movement and functional brain response. Front. Psychol. 11:587925. 10.3389/fpsyg.2020.587925 33519604 PMC7838443

[B80] Wilson R. S. Segawa E. Boyle P. A. Bennett D. A. (2012). Influence of late-life cognitive activity on cognitive health. Neurology 78, 1123–1129. 10.1212/WNL.0b013e31824f8c03 22491864 PMC3320053

